# Green Positive Guidance and Green Positive Life Counseling for Decent Work and Decent Lives: Some Empirical Results

**DOI:** 10.3389/fpsyg.2016.00261

**Published:** 2016-03-02

**Authors:** Annamaria Di Fabio, Ornella Bucci

**Affiliations:** Psychology Section, Department of Education and Psychology, University of FlorenceFirenze, Italy

**Keywords:** green positive guidance, green positive life counseling, green positive decent work, decent life, empathy, connectedness to nature

## Abstract

This article discusses green positive guidance and green positive life counseling for decent work and decent lives. From a green guidance perspective, the connectedness to nature construct is important both in terms of the meaning of work and life construction. The study discussed in this article analyzed the relationship between empathy and connectedness to nature, controlling for the effects of fluid intelligence and personality traits. In this connection, the Advanced Progressive Matrices, the Big Five Questionnaire, and the Interpersonal Reactivity Index were administered to 144 Italian high school students. The study revealed that connecteness to nature was not associated with fluid intelligence and was only moderately associated with personality traits. It was empathy that showed the highest association with connectedness to nature. The results open new opportunities for future research and interventions in green positive guidance/life counseling and green positive decent work.

## Introduction

Green guidance is concerned with sustainable development (Plant, 2013), environmental conscientiousness (Career Development Association of Australia [CDAA], 2010), and responsibility for the ecosystem (Barham and Eagleson, 2013). It deals with the choice of work and jobs that minimize environmental harm and that heighten awareness of the importance of green careers (Career Development Association of Australia [CDAA], 2010). It encourages individuals to consider the environmental implications of their career choices (Plant, 2013) and to balance work and other aspects of their lives (Guichard, 2013a; Plant, 2013), thereby sustaining a way of life that promotes health, economic security, and social justice (Career Development Association of Australia [CDAA], 2010). Green guidance also encourages organizations to pay more attention to sustainability issues and to recruit staff who understand these concerns on the one hand, and, on the other hand, it encourages job applicants to consider organizations that view their social responsibilities seriously and that implement enlightened human resource policies (Career Development Association of Australia [CDAA], 2010).

Green guidance focuses on sustainability issues, especially sustainability in career practices and career interventions aimed at promoting a balance between individual aspirations and societal and global needs (Guichard, 2013a; Plant, 2013). It thus underlines the importance of aligning individual needs with the needs of society, the environment, and the common good (Barham and Eagleson, 2013; Guichard, 2013a). It promotes ethical values in career guidance and counseling that contribute to building efficient, wealthier, and more just societies (Guichard, 2013a).

In relation to green guidance and career practice, the concept of ecological well-being can be used as a measure of how successful an ecological system is in managing, distributing, and sustaining environmental resources (Center on Education and Work [CEW], 2015). Green guidance focuses on ecological well-being that becomes a “natural” way of thinking, that is closely linked with people’s lives, and that is embedded in career guidance delivery (Barham and Eagleson, 2013).

A green challenge for career practitioners is how they engage their clients in considering the kind of world they want to live and the role they want to play in it (Guichard, 2013a; Plant, 2013). Clients are citizens in the present and have a role to play in constructing the future, which they can discuss with their career advisors (Guichard, 2013a; Plant, 2013).

Green guidance can also help move career practice away from the constraints of the dominant economic discourse toward a professional discourse that enables practitioners to assist clients achieve a fulfilling life in a more socially just, sustainable society (Barham and Eagleson, 2013). It is important to consider positive, individually satisfying ways of contributing to society not measured solely in monetary terms (Blustein, 2006, 2011).

Just as green guidance has to attend to wider ecological concerns, career practice has to take into consideration individual well-being, organizational well-being, community well-being, and the well-being of the planet. Individual well-being is concerned with responsibility for one’s own future, inclusion, and evaluation by society (Irving, 2013); psychological well-being (Kenny et al., 2014); adaptive functioning (Di Fabio and Kenny, 2015); and securing a sense of dignity and a livable wage (Blustein, 2006; Guichard, 2013a; Guichard and Di Fabio, 2015). Organizational well-being is concerned with the fulfillment of individual and organizational potential (Di Fabio, 2014a); effectiveness (ILO, 2015); productivity (ILO, 2015); customer and employee satisfaction (ILO, 2015); and workforce preparation (Di Fabio et al., in press). Community well-being is concerned with the fulfillment of individual and community potential (Wiseman and Brasher, 2008); connectedness (social networks) (Wiseman and Brasher, 2008); livability (infrastructures) (Wiseman and Brasher, 2008); and equity (values of diversity, social justice, and individual empowerment) (Wiseman and Brasher, 2008). Well-being of the planet is concerned with the achievement of an acceptable standard of living for all resulting from economic growth, capital accumulation, excess production, and unimpeded free market development (Irving, 2013). In terms of green guidance, career practice has to take these four kinds of well-being into consideration.

Green guidance also stresses the importance of the fundamental aims of career interventions (in line with the UNESCO Chair on Lifelong Guidance and Counseling, Guichard, 2013a) but from a “green” perspective, affirming the relevance of contributing to the good of humanity, contributing to the development of decent work in a sustainable and fair world economy (Guichard and Di Fabio, 2015), promoting more and better jobs and social inclusion (ILO, 2015), and reducing poverty (Di Fabio, 2014a). If “decent work” enhances sustainable development and sustainable careers (ILO, 2015), “green decent work” (Di Fabio, 2016) goes further by meeting the needs of the present without compromising the ability of future generations to meet their own needs (World Commission on Environment and Development [WCED], 1987) by achieving human development goals while preserving the resources of the environment and the ecosystem (Commission of the European Communities, 2009), by developing sustainable and green careers (Anand and Sen, 2000), by promoting a better quality of life and careers (International Institute for Sustainable Development [IISD], 2015). Green decent work (Di Fabio, 2016) also stresses the importance of connectedness with the natural environment (climate, resources, …) and environmental sustainability (Barham and Eagleson, 2013; Guichard, 2013a); the importance of the relational environment between people (Blustein, 2011) and social networks (Wiseman and Brasher, 2008); and ethical values (justice, equity, and fairness) (Barham and Eagleson, 2013).

A green positive guidance perspective (Di Fabio, 2016) can be introduced in respect of individual concerns as well as environmental concerns. Individual concerns are concerns for one’s own future in line with one’s own aims, and one’s own talents, potential, and relationships with significant others. Environmental concerns are concerns not only for one’s own future but for the future of others too and can be linked to fairness institutions, social justice, environmental well-being, beneficial exploitation of resources, and the preservation of a healthy planet and environment for future generations. Green positive guidance (Di Fabio, 2016) stresses the importance of designing one’s own future on the basis of sustainable development and careers, and also considering the future of others and of the planet.

Connectedness to nature is an important feature of green positive guidance and life counseling (Di Fabio, 2016) as the inclusion of nature in one’s representation of oneself is integral to the meaning of work (Blustein, 2006, 2011) and life construction (Guichard, 2013b).

Connectedness to nature was originally defined as “the extent to which an individual includes nature within his/her cognitive representation of self” (Schultz, 2002, p. 67). The definition later included the individual’s affective and experiential connection with nature (Mayer and Frantz, 2004). People who have such connectedness tend to perceive themselves as members of the broader natural world and community. They have a sense of affinity with it, considering themselves as belonging to the natural world as much as it belongs to them, and believing their well-being is linked to the well-being of the natural world (Mayer and Frantz, 2004).

Regarding environmental issues, there is a growing literature on ecological dimension of intelligence in terms of social development and cognitive patterns of human development (Gifford et al., 2011; Salahodjaev, 2016). A recent study (Salahodjaev, 2016) offers for example new statistical evidences of a negative link between intelligence and deforestation. Besides, the study underlines that psychological aspects such as intelligence are regularly ignored in statistical modeling of deforestation and focuses on intelligence as predictor of social and human capital (Salahodjaev, 2016) concerning environmental issues. Furthermore in previous studies individuals with higher IQ scores emerged more available both to favor cooperation and to have longtime horizon (Shamosh and Gray, 2008; Jones and Podemska, 2010). So, intelligence seems to be a psychological aspect we have to take into account to control its effect regarding environmental themes.

A green career choice (Bauer and Aiman-Smith, 1996) is updated for many reasons. We have to consider that organizations are “going green” highlighting as a proactive company interest for environment was also positively related to perceived company attractiveness, intentions to pursue employment with that company, and acceptance of a job offer. Individuals consider a “premium green” paying attention to environment al concerns, and willing to make changes in their lives in order to protect the environment (Fergnsen, 1993). Moreover in an environmental psychology perspective environmental issues as for example energy conservation, recycling, fresh water, and pollution are considered everyday commons dilemmas and intelligence and other personal characteristics have to take into consideration to resolve them (Gifford et al., 2011). On the other side, regarding personality, in previous studies a relation between personality traits and connectedness to nature emerged (Hirsh and Dolderman, 2007; Nisbet et al., 2008; Hirsh, 2010), showing in particular associations with agreeableness and openness. Very cooperative, friendly and generous individuals perceived themselves as more connect to nature (Hirsh and Dolderman, 2007; Nisbet et al., 2008; Hirsh, 2010). Also individuals who are interested in new and different experiences, opened to contact with different cultures and customs showed a highest connectedness to nature (Nisbet et al., 2008; Hirsh, 2010). These individuals have also a general sense of respect for nature and for other people (Nisbet et al., 2008).

Regarding the relationship between empathy and connectedness to nature, Schultz (2000) stresses concern for environmental issues. However, very few studies have actually analyzed this relationship (Berenguer, 2007; Cheng and Monroe, 2012; Liefländer et al., 2013). Berenguer (2007) shows the effect of empathy on pro-environmental attitudes and behavior, while Cheng and Monroe (2012) have found that children who enjoy nature generally have empathy for all living creatures. Liefländer et al. (2013) contend that an increase in such empathy will result in increased inclusion with nature. According to the previous considerations, the present study analyzed the relations between empathy and connectedness to nature, also controlling for the effects of fluid intelligence and personality traits. The relationship between empathy and connectedness to nature has not been studied extensively in the literature, and it has never been controlled for the effects of fluid intelligence and personality traits.

### Aim and Hypotheses

On the basis of the theoretical framework presented above, this study analyzed the relationship between empathy and connectedness to nature, controlling for the effects of fluid intelligence and personality traits on connectedness to nature. The following hypotheses were formulated.

H1: A relationship exists between empathy and connectedness to nature (Schultz, 2000; Berenguer, 2007; Cheng and Monroe, 2012; Liefländer et al., 2013).
H2: Empathy explains a significant percentage of the variance in connectedness to nature beyond that explained by the effects of fluid intelligence and personality traits.

## Materials and Methods

### Participants

The research participants were 144 high school students who were attending the last year of a high school in Tuscany. All the high school students in their final year of school were invited to participate in the study, of whom 94% agreed to participate. Sixty-six (45.80%) of the participants were males and 78 (54.20%) were females. The ages of the participants ranged from 18 to 20 years (*M* = 18.61, *SD* = 0.51).

### Measures

#### Advanced Progressive Matrices (APM)

The Advanced Progressive Matrices (APM) test by Raven (1962) in the Italian version by Di Fabio and Clarotti (2007) was used to evaluate fluid intelligence. The APM comprises two series of items: Series I has 12 items, and Series II has 36 items. Participants have to choose one of the alternative answers for each item. Each item has eight possible alternatives. The Cronbach’s alpha coefficient in the present study was 0.93.

#### Big Five Questionnaire (BFQ)

The Big Five Questionnaire (BFQ, Caprara et al., 1993) was used to evaluate personality traits. The questionnaire has 132 items with response options on a 5-point Likert scale ranging from 1 = *Absolutely false* to 5 = *Absolutely true*. The questionnaire measures five personality traits. The Cronbach’s alpha coefficients were: 0.82 for Extraversion (example of item: “I think that I am an active and vigorous person”), 0.76 for Agreeableness (example of item: “I understand when people need my help”), 0.83 for Conscientiousness (example of item: “I tend to be very thoughtful”), 0.91 for Emotional Stability example of item: (“I do not often feel tense”), and 0.77 for Openness (example of item: “I am always informed about what is happening in the world”).

#### Interpersonal Reactivity Index (IRI, Davis, 1980)

The Interpersonal Reactivity Index (IRI, Davis, 1980) in the Italian version by Albiero et al. (2006) was used to evaluate empathy. The IRI has 28 items with response options on a 4-point Likert scale ranging from 1 = *Never true* to 4 = *Always true*. The questionnaire measures four dimensions: Fantasy, Empathic concern, Perspective taking, Personal distress. The Cronbach’s alpha coefficients were: 0.83 for Fantasy (example of item: “I really get involved with the feelings of the characters in a novel”), 0.86 for Empathic concern (example of item: “I often have tender, concerned feelings for people less fortunate than me”, 0.84 for Perspective taking (example of item: “I believe that there are two sides to every question and try to look at them both”), 0.81 for Personal distress (example of item: “In emergency situations, I feel apprehensive and ill-at-ease”).

#### Connectedness to Nature Scale (CNS)

The Connectedness to Nature Scale (CNS, Mayer and Frantz, 2004) in the Italian version by Di Fabio (2016) was used to evaluate connectedness to nature. The scale has 14 items with response options on a 5-point Likert scale ranging from 1 = *Strongly agree* to 5 = *Strongly disagree*. Examples of items are: “I often feel a sense of oneness with the natural world around me”, “I think of the natural world as a community to which I belong”, “I have a deep understanding of how my actions affect the natural world”. The Cronbach’s alpha coefficient was 0.91.

### Procedure and Data Analysis

The administration of the instruments was carried out in groups in the classrooms by trained psychologists to counterbalance the administration sequence of the instruments to control for the possible effects of presentation. The administration adhered to the requirements of privacy and informed consent in Italian law (Law Decree DL-196/2003) and the ethical standards for research of the Declaration of Helsinki revised in Fortaleza (World Medical Association [WMA], 2013).

Descriptive statistics, Pearson’s *r* correlations, and hierarchical regressions were carried out. Gender differences were examined for the studied variables in separate regressions for gender but no differences emerged. For this reason, only regressions for the entire sample are reported in the results session.

## Results

Means, standard deviations, and correlations between the APM, BFQ, IRI, CNS are reported in **Table 1**. Significant correlations emerged of connectedness to nature with personality traits (particularly with Agreeableness) and with empathy (particularly with Fantasy and Empathic concern).

**Table 1:** Means, standard deviations, and correlations relative to fluid intelligence, personality traits, empathy dimensions, connectedness to nature.

	*M*	*SD*	1	2	3	4	5	6	7	8	9	10	11
1. Fluid intelligence	35.47	5.82	-										
2. Extraversion	75.98	10.54	0.10	-									
3. Agreeableness	76.74	8.69	0.05	0.07	-								
4. Conscientiousness	79.10	9.52	0.02	0.26**	0.11	-							
5. Emotional Stability	61.92	13.97	0.03	0.02	0.27**	0.04	-						
6. Openness	79.13	9.32	0.12	0.36**	0.35**	0.27**	0.10	-					
7. Fantasy	19.27	3.78	0.05	0.02	0.25**	0.17*	0.13	0.39**	-				
8. Empathic concern	23.51	3.22	0.01	0.09	0.23**	0.18*	0.05	0.31**	0.40**	-			
9. Perspective taking	13.48	2.48	0.12	0.08	0.34**	0.14	0.34**	0.32**	0.34**	0.30**	-		
10. Personal distress	18.45	2.34	0.01	0.04	0.01	0.13	0.12	0.02	0.11	0.16	0.10	-	
11. Connectedness to nature	45.74	7.94	0.08	0.03	0.32**	0.19*	0.07	0.28**	0.33**	0.43**	0.13	0.03	-

N = 144. *p < 0.05, **p < 0.01.

The results of hierarchical regression analysis with connectedness to nature as the criterion measure and with fluid intelligence at the first step, personality traits at the second step, and empathy dimensions at the third step are reported in **Table 2**.

**Table 2:** Hierarchical regression.

	Connectedness to nature
	β
*Step 1*	
Fluid intelligence	0.11
*Step 2*	
Extraversion	0.09
Agreeableness	0.28**
Conscientiousness	0.19*
Emotional stability	0.17*
Openness	0.10
*Step 3*	
Fantasy	0.32***
Empathic concern	0.42***
Perspective taking	0.12
Personal distress	0.05
*R^2^ Step 1*	0.01
*ΔR^2^ Step 2*	0.23***
*ΔR^2^ Step 3*	0.18***
*R^2^ total*	0.42***

*The contributions of Fluid intelligence (First step), Personality traits (Second step), and Empathy dimensions (Third step) to Connectedness to nature. *N* = 144. ^∗^*p* < 0.05, ^∗∗^*p* < 0.01, ^∗∗∗^*p* < 0.001.*

At the first step, fluid intelligence did not account for any variance in connectedness to nature (*R*^2^ = 0.01, n.s.). At the second step, personality traits accounted for 23% of the variance, and, at the third step, empathy dimensions accounted for 18% of the incremental variance. The regression model, in turn, accounted for 42% of the variance. The results show as empathy dimensions explain a significant percentage of the variance in connectedness to nature beyond that explained by personality traits. Fluid intelligence did not explain variance in connectedness to nature.

## Discussion

The study examined the relationship between empathy and connectedness to nature, controlling for the effects of fluid intelligence and personality traits on connectedness to nature. The two hypotheses of the study were confirmed as a relationship emerged between empathy and connectedness to nature (H1), also when taking into consideration the effects of fluid intelligence and personality traits (H2). It is important to state that fluid intelligence did not explain any of the variance in connectedness to nature. Even if relations between intelligence and environmental issues were substained in some studies (Jones and Podemska, 2010; Salahodjaev, 2016), fluid intelligence didn’t emerged related to connectedness to nature in this study. Regarding personality, in the present study connectedness to nature was associated to personality traits, particularly to Agreeableness. In line with the literature (Hirsh and Dolderman, 2007; Nisbet et al., 2008; Hirsh, 2010), individuals who are cooperative, friendly, and generous perceived themselves as more connect to nature and have also a general sense of respect for nature as well for other people. Furthermore the results relative to the second hypotheses showed, however, that empathy explained a significant percentage of the incremental variance with respect to personality traits in relation to connectedness to nature. The study thus highlighted the relationship between empathy and connectedness to nature in line with other findings reported in the literature (Schultz, 2000; Berenguer, 2007; Cheng and Monroe, 2012; Liefländer et al., 2013), underlining as individuals that perceived themselves more close to other people and being able to take the point of view of others, also perceived a greater connectedness to natural world. More specifically, the empathy dimensions most closely associated with connectedness to nature were Fantasy and Empathic concern. The participants with a higher tendency to identify themselves with characters in movies, novels, plays, and other fictional situations (Davis, 1980), and with greater feelings of warmth, compassion, and concern for others (Davis, 1980), seemed to have a higher connectedness to nature (Mayer and Frantz, 2004). The close association between empathy and connectedness to nature suggests that individuals sensitive toward other people could also be sensitive toward nature. This could mean that nature is, on the one hand, accorded human qualities, and, on the other hand, it represents something that needs to be taken into consideration in the construction of professional and personal lives.

Although the study clearly showed the relationship between empathy and connectedness to nature, its limitations need nevertheless to be noted. The participants in the study were a group of Italian high school students in the Tuscany region who were not necessarily representative of all Italian high school students. Future research should therefore include participants more representative of Italian high school students of the different geographical areas in Italy. Future studies should also include participants from other international contexts.

Despite the limitations of the present study, the findings add to and extend the literature because no relations emerged between fluid intelligence and connectedness to nature, relations emerged both between personality traits, particularly agreeableeness and connectedness to nature (Hirsh and Dolderman, 2007; Nisbet et al., 2008; Hirsh, 2010), and between empathy and connectedness to nature (Schultz, 2000; Berenguer, 2007; Cheng and Monroe, 2012; Liefländer et al., 2013) but emphaty was able to add a percentage of incremental variance with respect to personality traits in relation to connectedness to nature.

If the results of the present study are confirmed by future research, new intervention approaches could be introduced. It is widely recognized that empathy is a trait that can be increased through specific training (Herbek and Yammarino, 1990; Hatcher et al., 1994) and also in its relationship to connectedness to nature (Liefländer et al., 2013). It may therefore be possible to utilize empathy to promote connectedness to nature and also green positive guidance and green positive life counseling.

Connectedness to nature is an important element in the meaning of work (Blustein, 2006, 2011) and life construction (Guichard and Di Fabio, 2010; Guichard, 2013b) in terms of green positive guidance and green positive life counseling (Di Fabio, 2016). Green positive guidance endeavors to provide support to individuals regarding the construction of their own futures in line with their aims and authentic selves (Di Fabio, 2014c), the expression of their own talents and potential, the enhancement of positive relationships with significant others, and building individual strengths (Boyatzis et al., 2002, 2015; Boyatzis and Saatcioglu, 2008; Di Fabio and Palazzeschi, 2008a,b, 2009, 2012; Di Fabio and Blustein, 2010; Di Fabio and Kenny, 2012a,b; Di Fabio et al., 2012, 2013; Amdurer et al., 2014; Di Fabio, 2014b, 2015a; Di Fabio and Saklofske, 2014a,b; Kenny et al., 2014; Di Fabio, 2015b). At the same time, green positive guidance endeavors to respond to environmental concerns, fairness institutions, social justice, the environment, and the exploitation of resources (Guichard and Di Fabio, 2015; Di Fabio, 2016).

In this green positive guidance and counseling framework (Di Fabio, 2016), a new perspective is opening up in relation to decent work that could be considered green positive decent work (Di Fabio, 2016), that is, decent work not only for the self but also for others, not only for the present moment but also for the future (Guichard, 2013a) – work that is focused on resources, promoting the sustainability of nature and the environment for future generations too. These considerations can extend the ILO’s (2015) definition of decent work by adding that it cannot produce effects that are damaging to human life or the environment Guichard (2013a). The achievement of green positive decent work could be the real challenge for the 21st century.

## Author Contributions

AF conceptualized the study, choose the theoretical framework, chose measures, designed the questionnaire. OB helped in the collection of the data. AF and OB analyzed the data and wrote the methods and results. Then all authors wrote the paper together and read and revised the manuscript several times.

## Conflict of Interest Statement

The authors declare that the research was conducted in the absence of any commercial or financial relationships that could be construed as a potential conflict of interest.
